# Comprehensive bioinformatics analysis reveals common potential mechanisms, progression markers, and immune cells of coronary virus disease 2019 and atrial fibrillation

**DOI:** 10.3389/fcvm.2022.1027026

**Published:** 2022-10-24

**Authors:** Yang Lu, Ning Zhao, Yimei Du

**Affiliations:** ^1^Department of Cardiology, Tongji Medical College, Union Hospital, Huazhong University of Science and Technology, Wuhan, China; ^2^Research Center of Ion Channelopathy, Tongji Medical College, Union Hospital, Huazhong University of Science and Technology, Wuhan, China; ^3^Tongji Medical College, Union Hospital, Institute of Cardiology, Huazhong University of Science and Technology, Wuhan, China; ^4^Key Lab for Biological Targeted Therapy of Education Ministry and Hubei Province, Tongji Medical College, Union Hospital, Huazhong University of Science and Technology, Wuhan, China; ^5^Department of Cardiology, The First Affiliated Hospital of Zhengzhou University, Zhengzhou, China

**Keywords:** COVID-19, atrial fibrillation, progression markers, differentially expressed genes, bioinformatics

## Abstract

**Objectives:**

Atrial fibrillation (AF) is the most common arrhythmia in coronary virus disease 2019 (COVID-19) patients, especially in severe patients. A history of AF can exacerbate COVID-19 symptoms. COVID-19 Patients with new-onset AF have prolonged hospital stays and increased death risk. However, the mechanisms and targets of the interaction between COVID-19 and AF have not been elucidated.

**Materials and methods:**

We used a series of bioinformatics analyses to understand biological pathways, protein-protein interaction (PPI) networks, gene regulatory networks (GRNs), and protein-chemical interactions between COVID-19 and AF and constructed an AF-related gene signature to assess COVID-19 severity and prognosis.

**Results:**

We found folate and one-carbon metabolism, calcium regulation, and TFG-β signaling pathway as potential mechanisms linking COVID-19 and AF, which may be involved in alterations in neutrophil metabolism, inflammation, and endothelial cell function. We identified hug genes and found that NF-κb, hsa-miR-1-3p, hsa-miR-124-3p, valproic acid, and quercetin may be key regulatory molecules. We constructed a 3-gene signature consisting of ARG1, GIMAP7, and RFX2 models for the assessment of COVID-19 severity and prognosis, and found that they are associated with neutrophils, T cells, and hematopoietic stem cells, respectively.

**Conclusion:**

Our study reveals a dysregulation of metabolism, inflammation, and immunity between COVID-19 and AF, and identified several therapeutic targets and progression markers. We hope that the results will reveal important insights into the complex interactions between COVID-19 and AF that will drive novel drug development and help in severity assessment.

## Introduction

Coronary virus disease 2019 (COVID-19), caused by severe acute respiratory syndrome coronavirus 2 (SARS-CoV-2), has become a global pandemic (1). Currently, more than 500 million cases of COVID-19 have been diagnosed worldwide, with more than 6 million deaths (2). Although the majority of COVID-19 patients (81%) present with mild disease, more than 15% have developed severe disease to the point of multi-organ failure (1). The incidence of malignant arrhythmias and mortality were higher in COVID-19 patients with cardiac injury than in patients without cardiac injury (3).

Atrial fibrillation (AF) is a well-known cardiovascular risk factor and a cause of death in the general population (4). Studies have shown that among hospitalized COVID-19 patients, a history of AF leads to more severe clinical symptoms, higher mortality, and hospitalization events, which may be related to the underlying inflammatory status of AF patients (5). Importantly, AF is the most common arrhythmia in COVID-19 patients, occurring more frequently in critically ill patients (6). AF may cause embolism, hemodynamic disturbances, and increase the severity and mortality of COVID-19 (7). In critically care patients, AF independently increases the risk of the length of hospital stay, stroke, and death (8). It has been shown that alterations in endothelial cells and immune cells may be associated with the severity and complications of COVID-19 (9, 10). Endothelial cell tropism, detection and response, as well as disruption of vascular function, are prominent features of human COVID-19 infection (11, 12). Persistent immune dysregulation including altered neutrophil and macrophage function causes oxidative stress and altered endothelial cell function, which exacerbate the pathological process of COVID-19 (13–15). Although electrical and calcium processing and structural remodeling play key roles in AF pathophysiology, the underlying causes of AF in COVID-19 patients are largely unknown, making the management of AF patients during a pandemic particularly challenging. Therefore, it is necessary to explore possible pathophysiological links between COVID-19 and AF and identify potential therapeutic targets.

In this study, we used a systems bioinformatics approach to detect shared differentially expressed genes (DEGs) and associated pathways between COVID-19 and AF. The shared DEGs were used to identify protein-protein interaction (PPI) network, gene regulatory network (GRN), and protein-chemical interactions, and to construct AF-associated gene signatures for assessing the severity of COVID-19. Finally, these genes expression was localized by single-cell RNA sequencing (scRNA-seq) and they may be potential prognostic markers and therapeutic targets.

## Materials and methods

### Gene expression dataset

**FIGURE 1:**
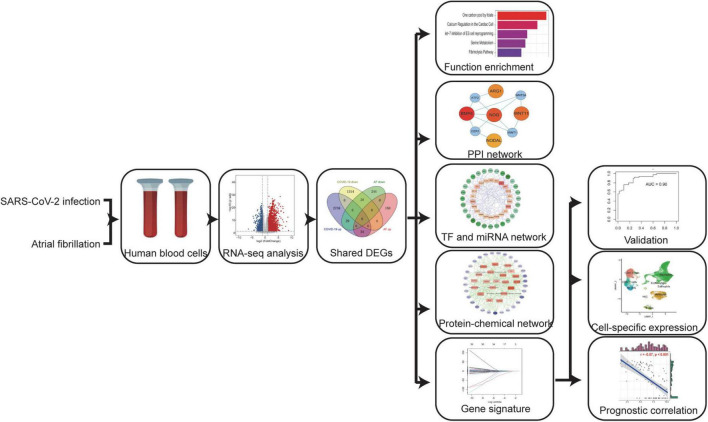
A systems bioinformatics approach was used to analyze the differentially expressed genes (DEGs) and associated pathways shared between COVID-19 and AF. Shared DEGs were used to identify protein-protein interaction (PPI) networks, gene regulatory networks (GRN), and protein-chemical interactions, and to construct AF-associated gene signatures to assess the severity of COVID-19. Localizing these genes expression by scRNA-seq and assessing their prognostic relevance.

The selected dataset details are shown in Table 1. The dataset GSE171110 (16) was obtained from Gene Expression Omnibus (GEO), including the peripheral blood transcriptional profiles by RNA-seq from 44 severe COVID-19 (80% male, median age 60 years) patients and 10 healthy controls, to obtain DEGs between COVID-19 patients and healthy controls. Dataset GSE75092 (17) contains peripheral blood transcriptional profiles by array from 3 paroxysmal AF patients and 3 healthy controls to analyze the DEGs of AF. The dataset GSE157103 (18) containing 102 different severity COVID-19 patients was used to construct the least absolute shrinkage and selection operator (LASSO) regression model and prognostic analysis. The dataset GSE152418 (19) containing 17 different severity COVID-19 patients was used to validate the model efficacy. Another validation dataset came from Aschenbrenner et al. (20), containing 39 COVID-19 patients, whose standardized count tables were obtained from the website FASTGenomics.^1^ The Bonn cohort (21) dataset was downloaded from FASTGenomics. This study included scRNA-seq data on fresh whole blood, fresh (peripheral blood mononuclear cells) PBMC, and frozen PBMC from 19 control donors and 22 COVID-19 patients. The details of the study design are shown in a flowchart (Figure 1).

**TABLE 1:** Datasets used.

Dataset	Disease	Case samples	Control samples	Usage here
GSE171110 (16)	COVID-19	44	10	Screening COVID-19 DEGs
GSE75092 (17)	AF	3	3	Screening AF DEGs
GSE157103 (18)	COVID-19	102	26	Construction of LASSO regression model; prognostic analysis
GSE152418 (19)	COVID-19	17	17	Model validation
Aschenbrenner et al. (20)	COVID-19	39	10	Model validation
Bonn cohort (21)	COVID-19	22	19	Single-cell sequencing analysis

### Identification of shared differentially expressed genes

The DEGs of RNA-seq dataset GSE171110 were identified using the “DESeq2” package in R (v 4.0.2), and the sample sizes of two groups meet the statistical requirements of “DESeq2” and no pretreatment is required. The “DESeq” function is used to analyze the differences between the two groups. The DEGs of microarray dataset GSE75092 were identified using the “limma” package. The “makeContrasts” function is used to compare the differences between the two groups. Both datasets use Benjamini-Hochberg correction to control the false discovery rate. Genes with *P*-value < 0.05 and | log2 fold change| > 1 were considered DEGs. The shared DEGs were acquired using an online VENN analysis tool Venny.^2^, v2.1.0.

### Signaling pathway and gene ontology enrichment analysis

Enrichment analysis is an important analytical task for classifying common biological processes and clarifying the association of gene sets with the pathways in which they are located. Signaling pathway and gene ontology (GO) biological processes enrichment were analyzed utilizing EnrichR.^3^ Enrichr uniquely integrates knowledge from many high-profile projects, providing various methods to calculate genomic enrichment. The shared DEGs were entered into Enrichr and visualize the results. Kyoto Encyclopedia of Genes and Genomes (KEGG), WikiPathways, Reactome, and BioCarta databases were used to specify the shared pathways between COVID-19 and AF. The top 10 GO terms and pathways of up and down DEGs were visualized in the bar plot.

### Protein-protein interaction analysis

PPI networks clarify protein-protein interrelationships and are the basis and key target for forming insights into cellular mechanisms. STRING database (version 11.0) was used to analyze PPI of shared DEG through NetworkAnalyst v3.0 Web server. The interactome with medium (400)—high (1,000) confidence score was selected. Then the PPI network was imported into the Cytoscape^4^ software. Cytohubba has 11 methods for studying networks from different perspectives, of which maximum cluster centrality (MCC) is the best The MCC algorithm in the CytoHubba plugin was used (v0.1) to calculate the top 10 hub genes.

### Gene regulatory network analysis

JASPAR is a publicly available resource for profiles of TFs from multiple species in six taxonomic groups. We use the NetworkAnalyst v3.0 platform to locate topologically plausible TFs from the JASPAR database, which are often bound to our mutual DEGs The R package “multiMiR” was used to predict miRNA-gene interactions. The multiMiR package includes 8 databases for predicting miRNA-target gene interactions, of which 3 are experimentally validated miRNA-target genes, which are the most comprehensive miRNA-target gene relationship prediction tools available. Only the experimentally verified interactions were included in the follow-up analysis. Cytoscape’s Cytohubba plugin was used to calculate the MCC scores and reserve the top 50 nodes of MCC scores.

### Protein-chemical compound analysis

Prediction of Protein-Chemical compound is one of the important parts in this study. Comparative Toxicogenomics database was used to identify protein–chemical interactions *via* Network Analyst. Comparative Toxicogenomics database provides manually curated information about chemical–gene/protein interactions, chemical–disease and gene–disease relationships. Cytoscape’s Cytohubba plugin was used to calculate the MCC scores and reserve the top 50 nodes of MCC scores.

### Construction of least absolute shrinkage and selection operator regression model

Lasso regression is a machine learning algorithm for feature filtering that avoids overfitting by selectively placing variables into the model to obtain better performance parameters. The “glmnet” package was used to perform LASSO regression. The shared DEGs were incorporated into the LASSO regression model, in which penalties were applied to DEGs for preventing the overfitting effects of the model. The dependent variable for critically patients was set to “1” and non-critically ill patients was set to “0.” The “binomial” regression type was select. The penalty parameter (λ) for the model was taken as lambda.1se and determined by 10-fold cross-validation. The “ROCR” package was used to calculate the receiver operating characteristic (ROC) curve.

### Analysis of single-cell RNA sequencing-seq dataset

The R package “Seurat” was used to process the expression matrix. Cells with more than 25% of mitochondrial reads, less than 250 expressed genes, or more than 5,000 genes with less than 500 transcripts detected were excluded from the analysis, and only those genes that appeared in more than 5 cells were considered for downstream analysis. After normalization of the matrix, 2000 high variable genes were selected by the “vst” method and the matrix was downscaled and clustered using the top 20 principal components. Cell type annotation was based on individual clustering results and combined with known marker gene expression. The “umap” algorithm was used to visualize cell groups, and the “VlnPlot” function was used to visualize gene expression.

### Correlation analysis

The R package “ggstatsplot” was used for “spearman” correlation analysis of gene expression and prognostic indicators.

### Statistics

For differentially expressed gene analysis, an adjusted *P* < 0.05 was considered statistically significant. For GO and signaling pathway enrichment analysis and spearman correlation analysis, and a *P* < 0.05 was considered statistically significant.

## Results

1. Identification of shared DEGs between COVID-19 and AF.

**FIGURE 2:**
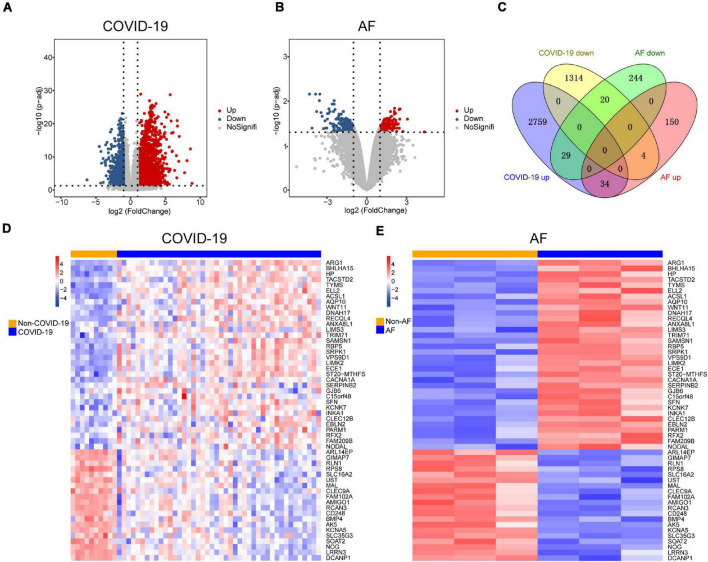
Comparison of transcriptome analysis of peripheral blood transcriptome reveals common genes shared by COVID-19 patients and AF patients. **(A)** The red dots and blue dots of the volcano map show the DEGs expressing rising and falling in COVID-19 patients, respectively. **(B)** The red dots and blue dots of the volcano map show the DEGs expressing rising and falling in AF patients, respectively. **(C)** Venn diagram shows the number of shared genes of COVID-19 and AF. **(D)** Heat map of shared genes expression in COVID-19 patients. **(E)** Heat map of shared genes expression in AF patients.

Since patients with severe COVID-19 have a high incidence of AF, The COVID-19 dataset (GSE171110) which contains 44 severe COVID-19 patients and 10 healthy controls was used to screen for DEGs. There were 4,160 DEGs in COVID-19 patients compared to healthy controls, of which 2,822 were up-regulated and 1,338 were down-regulated (Figure 2A). We identified 481 DEGs in the AF (GSE75092) dataset, of which 188 were up-regulated and 293 down-regulated (Figure 2B). Venn diagrams showed that AF patients shared 34 up-regulated genes and 20 down-regulated genes with COVID-19 patients (Figure 2C), for a total of 54 genes, which we identified as shared DEGs. The heat map showed the expression of these 54 shared genes in COVID-19 and AF, respectively (Figures 2D,E).

2. Functional enrichment analysis identified significant signaling pathways and GO terms.

**FIGURE 3:**
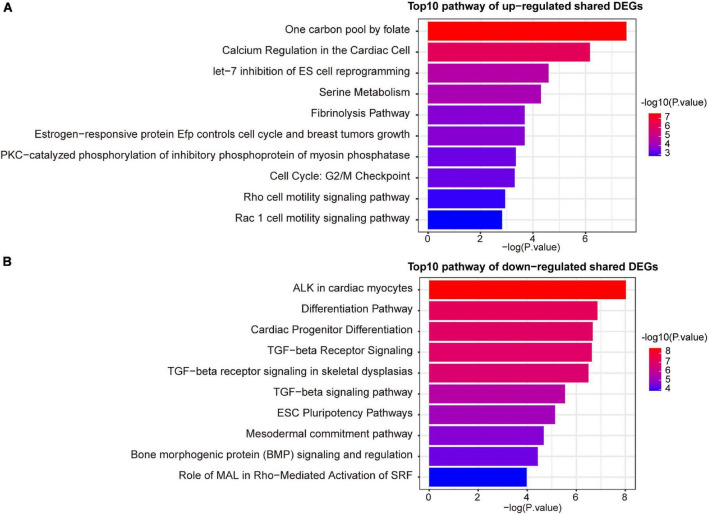
The top 10 signaling pathways of up-regulated **(A)** and down-regulated **(B)** shared DEGs.

**FIGURE 4:**
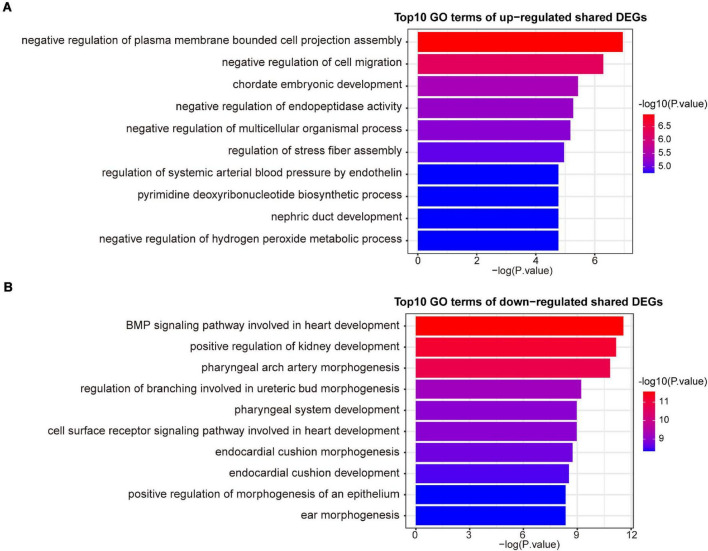
The top 10 GO biological processes terms of up-regulated **(A)** and down-regulated **(B)** shared DEGs.

A variety of signaling pathways and GO terms are involved in disease development. In this process, we used 34 up-regulated DEGs and 20 down-regulated DEGs to determine significant pathways and GO terms that may link COVID-19 and AF. The pathway analysis showed that the up-regulated DEGs were enriched in one carbon pool by folate, calcium regulation in the cardiac cell, pyrimidine biosynthesis, serine metabolism, and linoleic acid metabolism (Figure 3A). The down-regulated DEGs were enriched in ALK in cardiac myocytes, cardiac progenitor differentiation, and TGF-beta signaling pathway (Figure 3B). In addition, the GO biological processes enrichment analysis reveals that upregulated DEGs were primarily enriched in negative regulation of cell migration, pyrimidine deoxyribonucleotide biosynthetic process, and regulation of systemic arterial blood pressure by endothelin (Figure 4A). The downregulated DEGs were primarily enriched in the BMP signaling pathway involved in heart development and cell surface receptor signaling pathway involved in heart development (Figure 4B).

3. Construction of PPI network and identification of hub genes.

**FIGURE 5:**
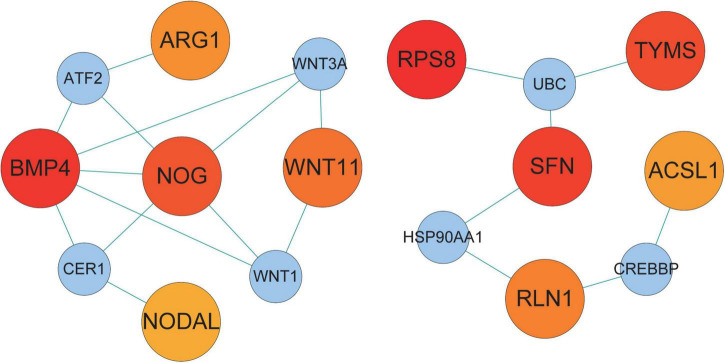
Hub protein network of shared DEGs in COVID-19 and AF. The connecting lines represent the presence of interactions between two nodes. The nodes in the network are divided into two sizes, with larger nodes representing the proteins encoded by DEGs and smaller nodes representing other proteins to distinguish them from DEGs. The color shades of the nodes indicate the degree of connectivity. 10 hub proteins are shown based on the number of interactions.

We examined the PPI network of STRING to predict the interactions of shared DEGs, and most of the interconnected nodes were considered to be hub genes. From the PPI network analysis of Cytoscape’s Cytohubba plugin, we listed the top 10 DEGs as the most influential genes (Supplementary Table 1). These hub genes are RPS8, BMP4, SFN, TYMS, NOG, AK5, WNT11, RLN1, ARG1, and ACSL1 (Figure 5). These hub genes can be potential biomarkers, which could also be the new therapeutic targets.

4. GRN analysis identified TF-DEGs and miRNA-DEGs interactions for the shared genes.

**FIGURE 6:**
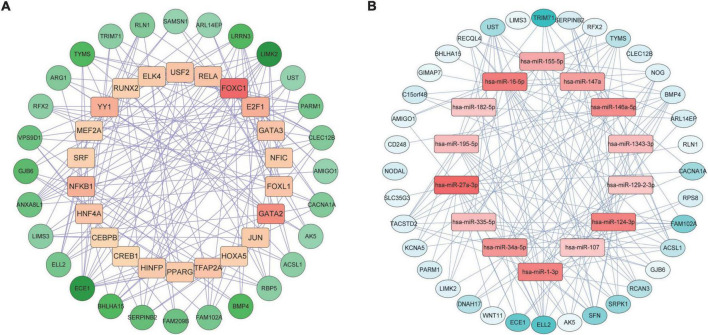
Gene regulatory network analysis of TF-DEGs **(A)** and miRNA-DEGs **(B)** in COVID-19 and AF. The circles represent DEGs and the squares represent TFs or miRNAs. nodes with top50 connectivity are shown, and nodes with high connectivity are presented in darker colors.

We constructed a TF-DEGs interaction network to find potential regulatory molecules. The circles in the figure represent DEGs, while the squares represent TFs. The color depth of the nodes depends on the number of connections between that node and other nodes in the network. Nodes with higher degrees are considered to be important hubs of the network. We identified transcription factors FOXC1, GATA2, NF-κb, YY1 as the more important transcription factors (Figure 6A). We also identified miRNA-DEG interaction networks, similar to previous analyses, with squares and circles representing miRNAs and genes, respectively. We found that has-miR-27a-3p, hsa-miR-1-3p, hsa-miR-124-3p, hsa-miR-34a-5p, and hsa-miR-146a-5p were the major regulators of DEGs (Figure 6B).

5. Protein-chemical interactions.

**FIGURE 7:**
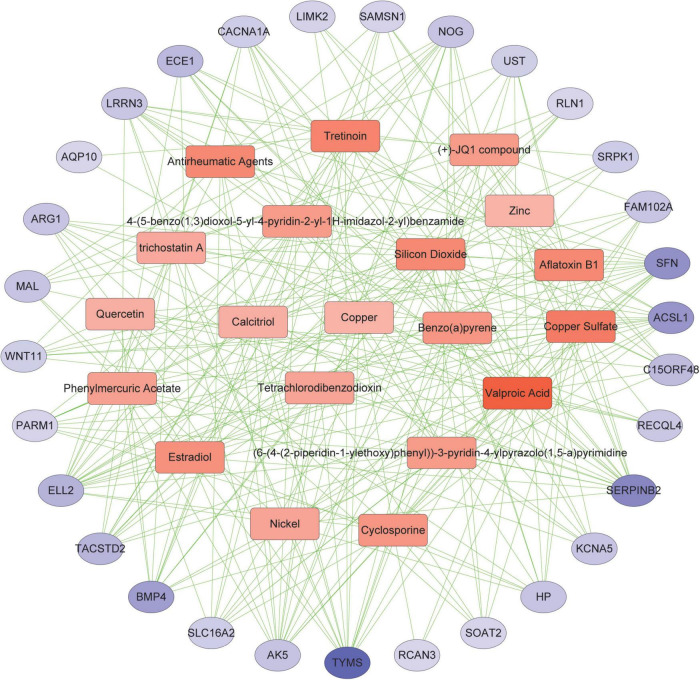
Association between COVID-19 and AF from the perspective of protein and chemical compounds. Circles represent proteins and squares represent compounds. nodes with top50 connectivity are shown, and nodes with high connectivity are presented in darker colors.

Protein-chemical interactions are an important study to understand protein function in intracellular biological processes, which may also contribute to drug discovery. We identified protein-chemical interactions that may affect shared DEGs. As shown, chemical compounds such as valproic acid, tretinoin, quercetin, estradiol, copper sulfate, benzo(a)pyrene, aflatoxin b1, etc. may act on shared DEGs such as TYMS, ACSL1, SERPINB2, and SFN (Figure 7).

6. Construction of an AF-associated gene signature to assess the severity of COVID-19.

**FIGURE 8:**
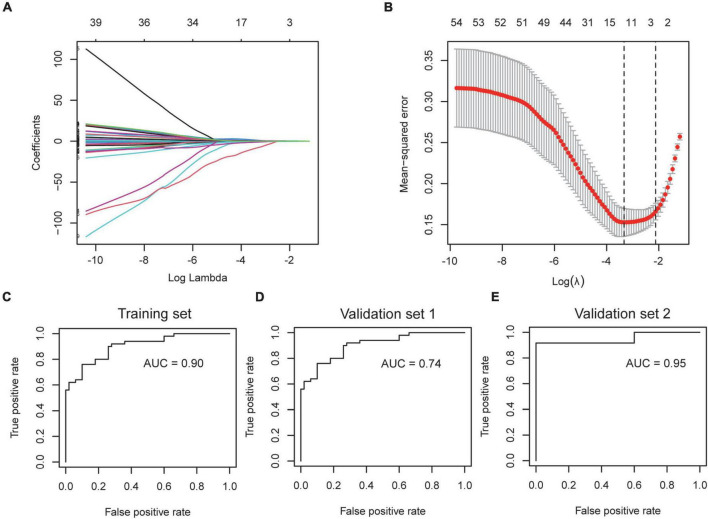
Construction and verification of severity assessment model. **(A,B)** The screened severity-related genes were incorporated into the Lasso regression model and penalties were applied for preventing the overfitting effects of the model. **(C)** The ROC curve of the training set used to distinguish the severity of COVID-19. **(D,E)** The ROC curve of validation set 1 and validation set 2 used to distinguish the severity of COVID-19.

The severity of COVID-19 needs to be accurately assessed to guide clinical decisions. Since the incidence of AF is associated with the severity of COVID-19, we wondered if the AF-associated genes could be used to stratify the condition of COVID-19 patients. We selected GSE157103 for the construction of the model, which contained the peripheral blood leukocyte transcriptome of 102 COVID-19 patients. The expression of shared DEGs was included in the LASSO regression model, with ICU status (ICU or no-ICU) as the response variable (Figure 8A). The most appropriate model was obtained when λ is 0.109 (Figure 8B), and ROC curve showed the AUC value is 0.90 (Figure 8C), with coefficients of 0.294, −0.357, and 0.169 for ARG1, GIMAP7, and RFX2, respectively. Subsequently, we used the Aschenbrenner et al.’s dataset (validation set 1, including 39 COVID-19 patients’ peripheral blood transcriptomes) to validate the model, and found it to have good accuracy (AUC = 0.74, Figure 8D). Due to the compositional similarities between leukocytes and PBMC, we wondered if the model could be used for datasets derived from PBMC. We validated the model on GSE152418 (validation set 2, containing 19 PBMC transcriptomes of COVID-19 patients) and found that the model also had high accuracy (AUC = 0.95) on PBMC samples (Figure 8E).

7. Cell-specific expression and prognostic predictive power of severity-associated genes.

**FIGURE 9:**
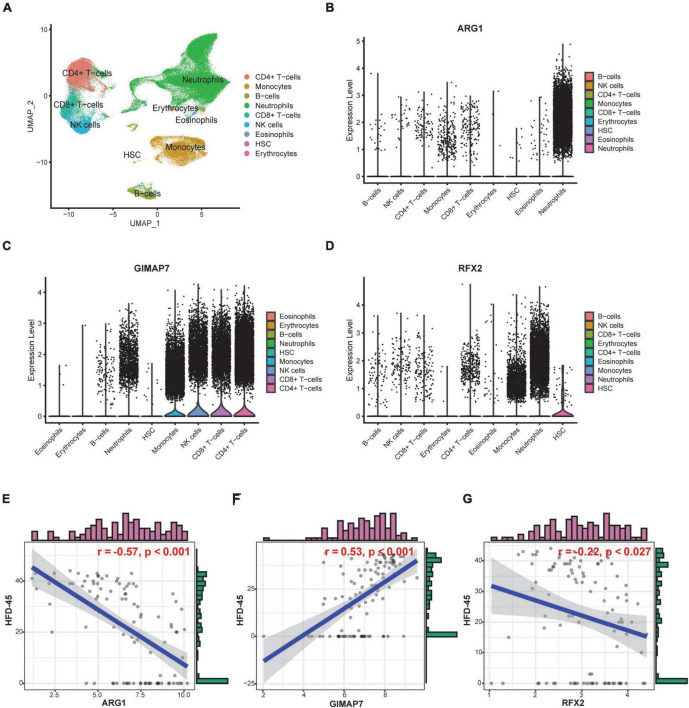
Cell-specific expression and prognostic predictive power of severity-associated genes. **(A)** UMAP plot shows 122,954 cells isolated from control and COVID-19 patients, colored by the main cell groups. **(B–D)** Violin plot of ARG1, GIMAP7, and RFX2 expression in different cell groups. The level of expression increases from left to right. **(E–G)** Correlation analysis of ARG1, GIMAP7, RFX2 expression levels and HFD-45.

To further explore the functional and clinical value of ARG1, GIMAP7, and RFX2, we analyzed the Bonn cohort, which included peripheral blood scRNA-seq data from 19 control and 22 COVID-19 patients. A total of 122,954 cells were divided into 9 subgroups, such as neutrophils, monocytes, and CD4^+^ T cells according to their marker genes (Figure 9A). We found that ARG1 was mainly expressed in neutrophils, GMAP7 was mainly expressed in CD4^+^ T cells and CD8^+^ T cells, and RFX2 was mainly expressed in hematopoietic stem cells (HSCs, Figures 9B–D), indicating that they may be involved in the function of these cell subgroups. Subsequently, we tested the prognostic value of severity-associated genes. The number of hospital-free days at day 45 (HFD-45) can reflect a composite of the length of stay and mortality, providing a refined clinical outcome. Patients with a length of stay longer than 45 days or who died during hospitalization are assigned a zero value, and patients with shorter lengths of stay and less severe illnesses are assigned a higher HFD-45 value. Correlation analysis showed that ARG1 (*r* = −0.57, *p* < 0.001) and RFX2 (*r* = −0.22, *p* < 0.027) were negatively correlated with HFD-45, while GIMAP7 (*r* = 0.53, *p* < 0.001) was significantly positively correlated with HFD-45 (Figures 9E–G), suggesting that they all had good prognostic predictive power.

## Discussion

In this study, by integrating COVID-19 and AF peripheral blood transcriptome, we identified 54 DEGs that are shared between COVID-19 and AF. DEGs were identified to be significantly enriched with GO terms and signaling pathways. A PPI network was constructed and 10 central genes were identified in PPI. TF-genes, miRNA-genes, and protein-chemical interactions revealed possible targets. A LASSO regression classification model composed of ARG1, GIMAP7, and RFX2 was constructed, which could be used to distinguish the severity of COVID-19. Finally, we found that ARG1, GIMAP7, and RFX2 were mainly expressed in neutrophils, T cells, and HSC, respectively. They were also associated with the prognosis of COVID-19. These findings may provide new insights into the mechanism of COVID-19 interaction with AF.

Multiple clinical studies have shown that patients with COVID-19 are prone to developing new AF and that new-onset AF predicts a poor prognosis (22–25). Pre-existing AF also exacerbates symptoms as well as the risk of death in patients with COVID-19 (26, 27). In addition, the incidence of AF is significantly higher in survivors of COVID-19 (28). This predicts a link between COVID-19 and the development of AF. There have been multiple attempts to intervene in the occurrence of both using medications (29, 30). The pathophysiology of SARS virus infection appears to be driven by an inflammatory immune response, with multiple markers of inflammation, such as C-reactive protein and the cytokine interleukin (IL)-6, which are associated with disease severity and mortality (31, 32). AF is also characterized by elevated inflammatory markers (33, 34). We aimed to explore possible pathophysiological interconnections between COVID19 and AF, including inflammation.

Signal pathways and GO terms can identify the biological processes of shared genes to determine the possible common pathological links between COVID-19 and AF. We have observed that some pathways have been previously shown to be related to COVID-19 and AF. For example, SARS-CoV-2 reshapes host folate and one-carbon metabolism after infection to support viral synthesis (35). Folate and one-carbon unit metabolism similarly supports the synthetic and secretory activities of immune cells (such as neutrophils) and may thereby induce AF (36). Calcium is essential for viral structure formation, entry, gene expression, viral maturation, and release (37). COVID-19 patients have increased calcium consumption and disturbed calcium concentrations (38, 39). We recently found that calcium disturbances may be an important pathological mechanism of inflammation causing new-onset AF (40). Meanwhile, AF-related calcium disorders leading to myocardial injury and apoptosis may exacerbate the COVID-19 inflammatory response (41). Therefore, calcium disorders may be one of the reasons why COVID-19 and AF exacerbate each other. Overdose and premature TGF-β response in COVID-19 patients may inhibit NK cell function and early control of the virus (42). In patients with AF, TGF-β promotes the production and maturation of cardiac collagen and the accumulation of extracellular matrix, changes atrial electrical conduction and excitability, and provides a basis for the maintenance of AF (43). In conclusion, these pathways may mediate the interaction of COVID-19/AF and become therapeutic targets.

We constructed PPI, TF-DEGs, miRNA-DEGs, and protein-chemical interaction networks for screening potential therapeutic targets against shared genes. PPI interaction network detected 10 hub gene signatures (RPS8, BMP4, SFN, TYMS, NOG, AK5, WNT11, RLN1, ARG1, and ACSL), which were associated with COVID-19 and AF and could be considered as candidates for prospective drug targets. The transcription factors NF-κb were found to be connected to shared DEGs. NF-κb signal promotes the production and signal transduction of tumor necrosis factor-α and interleukin-6 in COVID-19 (44). NF-κB also promotes the expression of cytokines and endothelial adhesion molecules during AF and down-regulates the cardiac sodium channel SCN5A to cause electrical remodeling (45–47). Thus modulation of NF-κB activation levels may reduce cytokine storm, decrease the severity of COVID-19, and reduce the occurrence of AF. We have determined that hsa-miR-1-3p and hsa-miR-124-3p were the most connected miRNA for shared DEGs. MiR-1-3p may regulate viral endocytosis, while SARS-CoV-2 uses endocytosis to enter host cells and induce COVID-19 (48, 49). MiR-1-3p also causes electrical remodeling of AF by targeting potassium channel genes (50). MiR-124-3p is upregulated during HIV-1 infection, causing downregulation of p21 and TASK1, consequently leading to increased release of viral particles (51). MiR-124-3p also rises in AF, regulates AXIN1, and promotes activation and proliferation of cardiac fibroblasts (52). In short, MiR-1 and miR-124 may serve as therapeutic targets for the simultaneous regulation of SARS-CoV-2 virus infection and AF-related myocardial remodeling. Then we identified the chemical compound valproic acid, a histone deacetylase inhibitor, as a modulator of DEG. Valproic acid can reduce the expression of angiotensin-converting enzyme 2 and transmembrane serine protease 2 required for SARS-CoV-2 virus entry (53), and reduces pathological atrial remodeling thereby preventing the development of AF, which may be a candidate for COVID-19 and AF therapy (54).

To date, several genetic markers have been available for assessing the severity of COVID-19. Considering the instability of a single gene, we constructed a robust model containing 3 genes, including ARG1, GIMAP7, and RFX2, using the LASSO Cox regression model as a screening method, and established a formula for AF gene-related COVID-19 severity score with a 0.90 AUC value. Subsequently, the assessment ability was validated in 2 independent cohorts from different platforms, which demonstrated the broad applicability of the genetic model. We also showed that ARG1, GIMAP7, and RFX2 are not only markers of severity, but also signs of hospital stay and death. Measuring these genes expression during the course of the disease and implementing interventions during the early stages may help track treatment response and prevent disease progression and poor prognosis.

Recent studies have shown that COVID-19 patients have reduced arginine levels and increased RNA expression of ARG1 in peripheral blood mononuclear cells (55, 56). Another study showed that circulating granulocyte-bone marrow-derived suppressor cells expressing high levels of arginase-1 (ARG1^+^ G-MDSC) were significantly increased in COVID-19 (57) which is consistent with our observations. Large amounts of Arg^+^ G-MDSC deplete plasma arginine, inhibit T cell proliferation and increase markers of endothelial cell dysfunction. Endothelial dysfunction is closely related to the development of AF (58, 59). Therefore, arginase 1 inhibition and/or arginine supplementation may be important for the prevention/treatment of severe COVID-19 and AF. Moreover, the protein-chemical interaction results showed that quercetin is a candidate for modulating arginase 1 activity, consistent with previous studies (60). Quercetin has shown therapeutic effects in influenza, and it is speculated that it may have therapeutic potential against COVID-19. Recent studies show that quercetin also ameliorates AF in rats (61). Therefore, quercetin may serve as a therapeutic target for modulating arginase 1 activity and treating COVID-19/AF.

The GIMAP gene family maintains the development of thymocytes and the survival of T cells in the periphery (62). Our results showed that GIPAM7 was expressed in CD4^+^ T cells and CD8^+^ T cells, and supported a better prognosis. In severe COVID-19, circulating T cells are severely reduced (63). T lymphocytes are the key coordinator of antiviral immune response by enhancing other the effector function of immune cell types or by directly killing infected cells (64). AF patients also have decreased lymphocytes, which is considered a sign of poor general health and physiological stress. Therefore, targeting GIMAP7 may reduce the extent of COVID-19 and AF by maintaining the number of lymphocytes. RFX2 has been reported in the past to control motile ciliogenesis in mice (65), and recently RFX2 was shown to promote survival in neutrophils (66). Our results showed that RFX2 was highly expressed in HSC compared to other immune cells. We speculate that the differentiation of HSC into granulocytes and monocytes may negatively affect COVID-19 and AF. It is important to examine whether RFX2 supports HSC survival and function in future studies.

To our knowledge, this is the first study to reveal the transcriptomic relationship between COVID-19 and AF. However, some limitations should be noted. First, confounding factors due to differences in sample size, sequencing platforms, and sample sources between the COVID-19 and AF datasets may cause bias and inaccuracy in the analysis results. Second, the relatively small size of the cohort may not be sufficient to capture all the key disease-associated genes needed to identify common DEGs. Third, further studies are needed to fully assess the biological function and clinical value of the potential targets identified in this work.

## Conclusion

In this study, we combined the transcriptomes of COVID-19 and AF to identify the mechanisms by which COVID-19 triggers AF and the interactions between them. We identified folate and one-carbon metabolism, calcium regulation, and TFG-β signaling pathway as potential mechanisms linking COVID-19 and AF. We performed transcriptional and post-transcriptional studies, constructed a PPI network, and found that NF-κb, hsa-miR-1-3p, hsa-miR-124-3p, valproic acid, and quercetin may be key regulatory molecules. We constructed a 3-gene signature consisting of ARG1, GIMAP7, and RFX2 for the assessment of COVID-19 severity and prognosis, and determined the cell-specific expression of these genes. This study provides new insights into underlying mechanisms and regulatory elements that may help develop potential novel drugs and assess patient severity to prevent the onset of severe COVID-19 and AF.

## Data availability statement

The datasets presented in this study can be found in online repositories. The names of the repository/repositories and accession number(s) can be found in the article/Supplementary material.

## Author contributions

YL conceived and designed the study, plotted the figures, and wrote the manuscript. NZ conducted data analysis and performed literature searches. NZ and YD reviewed and revised the manuscript. All authors contributed to the article and approved the submitted version.

## Footnotes

1
https://beta.fastgenomics.org/

2
https://bioinfogp.cnb.csic.es/tools/venny/

3
https://maayanlab.cloud/Enrichr/

4 https://cytoscape.org, v3.7.1.
