# Supplementary material for: Comprehensive bioinformatics analysis reveals common potential mechanisms, progression markers, and immune cells of coronary virus disease 2019 and atrial fibrillation
Source: Front Cardiovasc Med. 2022 Oct 24;9:1027026. doi: 10.3389/fcvm.2022.1027026 (PMC9637541; doi:10.3389/fcvm.2022.1027026)
Supplement: Supplementary file 1 [file Table_1.DOCX]

Supplementary Material

| node_name | MCC | DMNC | MNC | Degree | EPC | BottleNeck | EcCentricity | Closeness | Radiality | Betweenness | Stress | ClusteringCoefficient |
| --- | --- | --- | --- | --- | --- | --- | --- | --- | --- | --- | --- | --- |
| RPS8 | 167 | 0 | 1 | 167 | 129.548 | 545 | 0.11111 | 253.325 | 9.33088 | 150788 | 975044 | 0 |
| BMP4 | 123 | 0.10653 | 23 | 102 | 105.364 | 545 | 0.14286 | 207.4429 | 9.11029 | 155634.5 | 1534422 | 0.00427 |
| SFN | 73 | 0 | 1 | 73 | 126.421 | 545 | 0.14286 | 213.3476 | 9.80147 | 197724.4 | 2061736 | 0 |
| TYMS | 50 | 0 | 1 | 50 | 92.831 | 85 | 0.11111 | 180.3583 | 9.03309 | 82376.6 | 1239682 | 0 |
| NOG | 49 | 0.10653 | 23 | 28 | 105.216 | 6 | 0.125 | 155.0952 | 8.29963 | 6761.875 | 29274 | 0.0582 |
| WNT11 | 36 | 0 | 1 | 36 | 87.628 | 45 | 0.11111 | 135.4544 | 7.46691 | 45719.67 | 846706 | 0 |
| RLN1 | 29 | 0 | 1 | 29 | 75.261 | 44 | 0.11111 | 137.6798 | 8.13235 | 44589 | 517764 | 0 |
| ARG1 | 18 | 0 | 1 | 18 | 58.101 | 17 | 0.11111 | 118.8353 | 7.25 | 17137 | 138258 | 0 |
| ACSL1 | 17 | 0 | 1 | 17 | 22.711 | 17 | 0.09091 | 98.49845 | 6.25735 | 17136 | 193200 | 0 |
| NODAL | 13 | 0 | 1 | 13 | 86.419 | 8 | 0.11111 | 114.302 | 7.19853 | 7575 | 176332 | 0 |
| UBC | 3 | 0 | 1 | 3 | 105.222 | 86 | 0.125 | 201.3929 | 9.71875 | 76948.2 | 877092 | 0 |
| CER1 | 3 | 0.30779 | 2 | 3 | 86.383 | 1 | 0.125 | 139.0619 | 8.15257 | 1590.936 | 35344 | 0.33333 |
| ATF2 | 3 | 0.30779 | 2 | 3 | 81.162 | 1 | 0.125 | 141.3119 | 8.18566 | 9300.962 | 75412 | 0.33333 |
| WNT3A | 3 | 0.30779 | 2 | 3 | 85.323 | 1 | 0.125 | 146.1952 | 8.2886 | 9316.286 | 175500 | 0.33333 |
| WNT1 | 3 | 0.30779 | 2 | 3 | 87.809 | 1 | 0.125 | 146.1952 | 8.2886 | 9316.286 | 175500 | 0.33333 |

**Supplementary table 1.** The top 10 DEGs and other proteins rank according to cytoHubba algorithms.
